# Risk factors for lower limb deep vein thrombosis in patients with intracerebral hemorrhage: a retrospective study using LASSO regression to develop a nomogram

**DOI:** 10.3389/fneur.2026.1754883

**Published:** 2026-05-25

**Authors:** Xiuxiu Feng, Long Zhang, You Ling, Bing Zhong

**Affiliations:** 1 Department of Ultrasound, Yuebei People’s Hospital of Shantou University Medical College, Shaoguan, China; 2 Department of Neurosurgery, Yuebei People’s Hospital of Shantou University Medical College, Shaoguan, China

**Keywords:** cerebral hemorrhage, influencing factors, LASSO regression, lower limb deep venous thrombosis, nomogram

## Abstract

**Objective:**

To explore the influencing factors of clinically suspected lower limb deep vein thrombosis (DVT) in patients with cerebral hemorrhage and establish a nomogram clinical prediction model based on Lasso regression.

**Methods:**

A total of 297 patients with cerebral hemorrhage treated in our hospital from January 2023 to July 2025 were retrospectively included. They were randomly divided into the training group (*n* = 208) and the validation group (*n* = 89) in a ratio of 7:3. The training group was separated into the non DVT group (*n* = 156) and the DVT group (*n* = 52) based on the occurrence of lower limb DVT during hospitalization. Lasso regression analysis and multivariate logistic regression analysis were applied to screen the influencing factors of clinically suspected lower extremity DVT in patients with intracerebral hemorrhage. The nomogram was created based on the results of regression analysis. Calibration curves, receiver operating characteristic (ROC) curves, and clinical decision curve analysis (DCA) curves were plotted to evaluate the calibration, discrimination, and clinical utility of the nomogram model.

**Results:**

There was no statistical difference in baseline data between the training group and the validation group (*p* > 0.05). The results of Lasso and logistic regression analyses showed that Age (OR: 1.206), albumin (OR: 0.747), D-dimer (OR: 1.992), and the TyG index (OR: 2.061) were influencing factors of clinically suspected lower limb DVT in patients with cerebral hemorrhage (*p* < 0.05). The calibration curves of the training group and the validation group showed that the Hosmer-Lemeshow *χ^2^* statistic was 6.482 and 7.935, respectively, with *p* = 0.627 and 0.492 > 0.05, indicating that the model had good calibration. ROC curve showed that the AUC was 0.891 (95%CI: 0.845 ~ 0.937) and 0.848 (95%CI: 0.791 ~ 0.905), and the model had good discrimination. In addition, DCA curve showed that the model provided favorable net clinical benefits within the high-risk thresholds of 0.07–0.89 and 0.09–0.69.

**Conclusion:**

The nomogram prediction model constructed based on the influencing factors of this study has a certain predictive performance and can assist clinicians in assessing the risk of occurrence of clinically suspected lower limb DVT in patients with cerebral hemorrhage, but it should be considered preliminary before external validation is completed.

## 1 Introduction

Deep venous thrombosis (DVT) of the lower extremities is a common and severe complication in patients with intracerebral hemorrhage, and prophylactic anticoagulation is an effective strategy to reduce the incidence of lower extremity DVT (1). However, due to concerns that anticoagulant therapy may increase the risk of recurrent intracerebral hemorrhage, anticoagulation in critically ill patients with intracerebral hemorrhage is often delayed or withheld, posing a major challenge to subsequent treatment (2). Early identification of high-risk populations for lower extremity DVT and the implementation of appropriate interventions are crucial. Current thromboembolism risk assessment tools include the Padua prediction score, the stroke-associated VTE risk score, the Caprini score, and the Wells score. These tools are mostly applied in mixed populations and have not been clearly validated in patients with isolated intracerebral hemorrhage (3, 4). Moreover, the above scoring systems are primarily composed of static models, which fail to account for the dynamic nature of DVT risk in patients with intracerebral hemorrhage. Therefore, the search for new assessment methods for lower extremity DVT is necessary. This study aims to further clarify the early warning role of different clinical risk factors in the occurrence and development of clinically suspected lower extremity DVT after intracerebral hemorrhage and to preliminarily establish a nomogram clinical prediction model to guide anticoagulation therapy following intracerebral hemorrhage.

## 2 Study subjects and methods

### 2.1 Study subjects

Missing data were assessed using Little’s MCAR test, and the result showed *p* > 0.05, supporting the MCAR assumption, indicating that the data were missing completely at random; therefore, 8 cases were directly excluded using listwise deletion. A total of 297 patients with intracerebral hemorrhage treated in our hospital from January 2023 to July 2025 were retrospectively enrolled as study subjects. The study population was randomly divided into a training group (*n* = 208) and a validation group (*n* = 89) at a ratio of 7:3.

**Figure 1:**
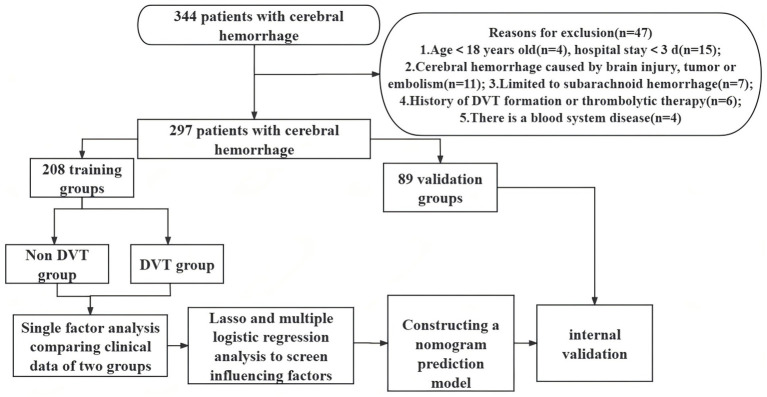
Case collection process diagram.

Inclusion criteria: (1) Meeting the criteria for spontaneous intracerebral hemorrhage (5), with diagnosis confirmed by computed tomography or magnetic resonance imaging at admission; (2) Age ≥18 years, with complete clinical data; (3) Hospitalization duration ≥3 days; (4) The bleeding sites included subdural hemorrhage, intraventricular hemorrhage, basal ganglia hemorrhage, and thalamic hemorrhage. Exclusion criteria: (1) Intracerebral hemorrhage caused by trauma, tumor, or embolism; (2) Lobar hemorrhage and subarachnoid hemorrhage; (3) History of DVT formation or thrombolytic therapy; (4) DVT following cerebral infarction; (5) Concomitant hematologic diseases. The case collection flowchart is shown in Figure 1.

### 2.2 Methods

#### 2.2.1 Lower extremity DVT assessment

The diagnosis of DVT followed the Guidelines for the Diagnosis and Treatment of Deep Venous Thrombosis (6), mainly based on clinical evaluation and confirmed by imaging. Specifically, if patients presented with signs or symptoms suggestive of DVT formation, such as limb swelling, pain, or a positive Homan’s sign, lower extremity Doppler ultrasonography was performed to confirm the presence of thrombosis. The 208 patients in the training group were divided into a non-DVT group (*n* = 156) and a DVT group (*n* = 52) according to the occurrence of lower extremity DVT during hospitalization. During hospitalization, 18 patients in the non-DVT group and 9 patients in the DVT group died, and all deaths were due to neurological causes.

#### 2.2.2 Clinical data collection

Clinical data were collected and recorded. Demographic characteristics included age, sex, smoking, and alcohol consumption. Admission and post-admission data included body mass index (BMI), hypertension, diabetes, coronary artery disease, admission systolic blood pressure, admission diastolic blood pressure, site of intracerebral hemorrhage, midline shift, admission National Institutes of Health Stroke Scale (NIHSS) score, Glasgow Coma Scale (GCS) score, prophylactic use of low-dose subcutaneous heparin, surgical approach, surgery duration, and bleeding volume. Laboratory tests included albumin, hemoglobin, D-dimer, lymphocyte count, neutrophil count, platelet count, leukocyte count, C-reactive protein, and the TyG index. The TyG index was calculated as ln [fasting triglycerides (mg/dL) × fasting blood glucose (mg/dL)]/2.

#### 2.2.3 Model construction and validation

(1) Data splitting: To construct and validate the prediction model, the entire cohort of 297 patients with cerebral hemorrhage was first randomly divided into a training group and an independent validation group at a ratio of 7:3. (2) Model construction: Based only on the data from the training group, the occurrence of lower limb DVT was used as the dependent variable. Variables with *p* < 0.05 in the univariate analysis were included in the LASSO regression analysis. Variables selected by LASSO regression with *p* < 0.05 were further included in the multivariate logistic regression analysis to identify independent predictors and construct the prediction model. (3) Preliminary evaluation of model performance in the validation group: The final model constructed in the training set was directly applied to the independent validation group to evaluate its generalization performance in new samples that had not been used during model development. (4) Stability assessment of model performance using Bootstrap internal validation: After obtaining the preliminary performance indicators, such as the AUC, in the training and validation groups, Bootstrap resampling was further applied separately in the training and validation groups to assess the stability of the model and correct for potential bias caused by the limited sample size, thereby verifying the robustness of the model.

### 2.3 Statistical analysis

Statistical analysis was performed using R 4.5.1 and SPSS 26.0. Categorical data were expressed as [*n* (%)] and analyzed with the χ^2^ test. Continuous data, all of which were normally distributed according to the Kolmogorov–Smirnov test, were expressed as mean ± standard deviation and analyzed with the independent-sample *t* test. Variables with *p* < 0.05 in univariate analysis were included in further analysis. All variables were assessed for multicollinearity, and the results showed that all VIF values were <10, indicating no multicollinearity; therefore, they could be included simultaneously in the regression analysis model. In addition, restricted cubic splines combined with the likelihood ratio test were used to determine whether there was a nonlinear association between each variable and Logit(P). The results showed that all *p* values were >0.05; therefore, all variables were considered as linear terms and included in the final model. Lasso regression analysis and multivariate logistic regression analysis were applied to screen for influencing factors of clinically suspected lower extremity DVT in patients with intracerebral hemorrhage. Based on the regression analysis results, a nomogram was constructed, and internal validation was performed using the bootstrap method. Calibration curves were plotted to evaluate the calibration of the nomogram model, receiver operating characteristic (ROC) curves were plotted to assess the discrimination of the model, and decision curve analysis (DCA) curves were drawn to evaluate its clinical utility. A *p* value <0.05 was considered statistically significant.

## 3 Results

### 3.1 Comparison of baseline data between training and validation groups

There were no statistically significant differences between the training and validation groups in age, sex, smoking, alcohol consumption, BMI, hypertension, diabetes, coronary artery disease, admission systolic blood pressure, admission diastolic blood pressure, site of intracerebral hemorrhage, midline shift, admission NIHSS score, GCS score, prophylactic use of low-dose subcutaneous heparin, surgical approach, surgery duration, bleeding volume, albumin, hemoglobin, D-dimer, lymphocyte count, neutrophil count, platelet count, leukocyte count, C-reactive protein, or TyG index (*p* > 0.05) (see Table 1).

**Table 1:** Comparison of baseline data between training group and validation group [*n* (%)/(*x̄* ± *s*)].

Index	Training group (*n* = 208)	Validation group (*n* = 89)	*χ* ^2^ */t*	*P*
Age (years)	57.40 ± 9.58	55.48 ± 9.95	1.564	0.119
Gender [*n* (%)]			0.490	0.484
Female	89 (42.79)	42 (47.19)		
Male	119 (57.21)	47 (52.81)		
Smoking [*n* (%)]	110 (52.88)	44 (49.44)	0.297	0.586
Drinking [*n* (%)]	32 (15.38)	18 (20.22)	1.043	0.307
BMI (kg/m^2^)	22.75 ± 2.49	22.31 ± 2.92	1.323	0.187
Hypertension [*n* (%)]	69 (33.17)	21 (23.60)	2.707	0.100
Diabetes [*n* (%)]	26 (12.50)	15 (16.85)	0.993	0.319
Coronary heart disease [*n* (%)]	36 (17.31)	12 (13.48)	0.673	0.412
Admission systolic blood pressure (mmHg)	151.05 ± 19.63	149.88 ± 20.10	0.467	0.641
Admission diastolic blood pressure (mmHg)	88.90 ± 14.66	87.15 ± 15.04	0.935	0.350
Location of cerebral hemorrhage [*n* (%)]			1.185	0.757
Subdural hemorrhage	66 (31.73)	26 (29.21)		
Intraventricular hemorrhage	49 (23.56)	24 (26.97)		
Basal ganglia hemorrhage	52 (25.00)	25 (28.09)		
Thalamic hemorrhage	41 (19.71)	14 (15.73)		
Midline shift [*n* (%)]	39 (18.75)	16 (17.98)	0.025	0.875
Admission NIHSS score (points)			1.442	0.230
≤15	130 (62.50)	49 (55.06)		
>15	78 (37.50)	40 (44.94)		
GCS score (points)			3.242	0.072
≥12	144 (69.23)	52 (58.43)		
<12	64 (30.77)	37 (41.57)		
Prophylactic use of low-dose subcutaneous heparin [*n* (%)]	45 (21.63)	23 (25.84)	0.625	0.429
Surgical approach [*n* (%)]			0.029	0.865
Minimally invasive surgery	140 (67.31)	59 (66.29)		
Craniotomy	68 (32.69)	30 (33.71)		
Operation time (h)			1.926	0.165
≤2	87 (41.83)	45 (50.56)		
>2	121 (58.17)	44 (49.44)		
Blood loss (mL)	23.14 ± 4.98	24.09 ± 5.66	1.445	0.150
Albumin (g/L)	36.58 ± 6.15	36.17 ± 5.73	0.537	0.592
Hemoglobin (g/L)	155.70 ± 38.64	151.96 ± 40.12	0.755	0.451
D-dimer (μg/mL)	1.10 ± 0.25	1.15 ± 0.26	1.560	0.120
Lymphocyte count (×10^9^/L)	1.09 ± 0.23	1.11 ± 0.25	0.669	0.504
Neutrophil count (×10^9^/L)	6.60 ± 1.48	6.67 ± 1.56	0.367	0.714
Platelet count (×10^9^/L)	154.72 ± 37.56	152.93 ± 38.95	0.372	0.710
White blood cell count (×10^9^/L)	7.95 ± 2.10	7.90 ± 2.03	0.190	0.850
C-reactive protein (mg/L)	16.40 ± 4.09	17.18 ± 4.16	1.498	0.135
TyG index	8.29 ± 0.62	8.31 ± 0.65	0.251	0.802

### 3.2 Univariate analysis of clinically suspected lower extremity DVT occurrence in patients with intracerebral hemorrhage in the training group

No statistically significant differences were found between the non-DVT group and the DVT group in terms of sex, smoking, alcohol consumption, BMI, diabetes, coronary artery disease, admission systolic blood pressure, admission diastolic blood pressure, site of intracerebral hemorrhage, midline shift, prophylactic use of low-dose subcutaneous heparin, surgical approach, surgery duration, hemoglobin, lymphocyte count, neutrophil count, platelet count, or leukocyte count (*p* > 0.05). Compared with the non-DVT group, the DVT group had higher values of age, bleeding volume, D-dimer, C-reactive protein, TyG index, a higher proportion of hypertension, admission NIHSS score >15, and GCS score <12, while albumin levels were lower (*p* < 0.05) (see Table 2).

**Table 2:** Univariate analysis of clinically suspected lower limb DVT in patients with cerebral hemorrhage in the training group [*n* (%)/(*x̄* ± *s*)].

Index	Non DVT group (*n* = 156)	DVT group (*n* = 52)	χ^2^*/t*	*P*
Age (years)	54.78 ± 9.24	65.25 ± 9.75	6.979	<0.001
Gender [*n* (%)]			0.321	0.571
Female	65 (41.67)	24 (46.15)		
Male	91 (58.33)	28 (53.85)		
Smoking [*n* (%)]	85 (54.49)	25 (48.08)	0.643	0.423
Drinking [*n* (%)]	22 (14.10)	10 (19.23)	0.788	0.375
BMI (kg/m^2^)	22.69 ± 2.74	22.91 ± 2.30	0.521	0.603
Hypertension [*n* (%)]	45 (28.85)	24 (46.15)	5.270	0.022
Diabetes [*n* (%)]	18 (11.54)	8 (15.38)	0.527	0.468
Coronary heart disease [*n* (%)]	25 (16.03)	11 (21.15)	0.717	0.397
Admission systolic blood pressure (mmHg)	150.49 ± 18.26	152.74 ± 20.08	0.750	0.454
Admission diastolic blood pressure (mmHg)	88.62 ± 14.13	89.75 ± 14.80	0.494	0.622
Location of cerebral hemorrhage [*n* (%)]			0.301	0.960
Subdural hemorrhage	49 (31.41)	17 (32.69)		
Intraventricular hemorrhage	38 (24.36)	11 (21.15)		
Basal ganglia hemorrhage	38 (24.36)	14 (26.92)		
Thalamic hemorrhage	31 (19.87)	10 (19.24)		
Midline shift [*n* (%)]	26 (16.67)	13 (25.00)	1.778	0.182
Admission NIHSS score (points)			7.904	0.005
≤15	106 (67.95)	24 (46.15)		
>15	50 (32.05)	28 (53.85)		
GCS score (points)			5.898	0.015
≥12	115 (73.72)	29 (55.77)		
<12	41 (26.28)	23 (44.23)		
Prophylactic use of low-dose subcutaneous heparin [*n* (%)]	35 (22.44)	10 (19.23)	0.236	0.627
Surgical approach [*n* (%)]			1.864	0.172
Minimally invasive surgery	109 (69.87)	31 (59.62)		
Craniotomy	47 (30.13)	21 (40.38)		
Operation time (h)			3.484	0.062
≤2	71 (45.51)	16 (30.77)		
>2	85 (54.49)	36 (69.23)		
Blood loss (mL)	21.97 ± 4.56	26.62 ± 6.91	5.541	<0.001
Albumin (g/L)	38.42 ± 6.92	31.05 ± 4.28	7.226	<0.001
Hemoglobin (g/L)	156.72 ± 37.27	152.64 ± 41.08	0.666	0.506
D-dimer (μg/mL)	1.03 ± 0.23	1.32 ± 0.28	7.443	<0.001
Lymphocyte count (×10^9^/L)	1.08 ± 0.23	1.12 ± 0.25	1.062	0.289
Neutrophil count (×10^9^/L)	6.59 ± 1.46	6.62 ± 1.58	0.126	0.900
Platelet count (×10^9^/L)	152.98 ± 38.45	159.94 ± 36.71	1.143	0.254
White blood cell count (×10^9^/L)	7.92 ± 2.12	8.04 ± 2.06	0.356	0.722
C-reactive protein (mg/L)	15.73 ± 3.96	18.42 ± 4.38	4.130	<0.001
TyG index	8.10 ± 0.69	8.89 ± 0.56	7.473	<0.001

### 3.3 Variable selection based on Lasso regression analysis

**Figure 2:**
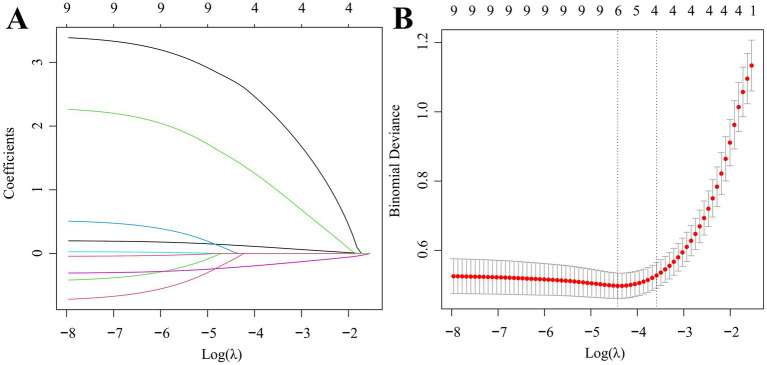
Lasso regression analysis feature variable selection process. **(A)** Lasso coefficient paths for predictors of suspected lower limb DVT in spontaneous cerebral hemorrhage patients. **(B)** Cross-validation deviance curves for Lasso model.

Tenfold cross-validation of Lasso regression determined the optimal parameter λ, and the λ.min value corresponding to the minimum likelihood deviance was chosen. At this λ value, the model included four non-zero coefficients: age, albumin, D-dimer, and TyG index. The Lasso variable selection process is shown in Figure 2.

### 3.4 Variable selection based on multivariate logistic regression analysis

The variables selected by Lasso regression were included as independent variables in the multivariate logistic regression analysis, with the occurrence of lower extremity DVT as the dependent variable. Variable assignment is shown in Table 3. Multivariate logistic regression analysis indicated that age (OR: 1.206, 95% CI: 1.111 ~ 1.308), albumin (OR: 0.747, 95% CI: 0.667 ~ 0.836), D-dimer (OR: 1.992, 95% CI: 1.066 ~ 2.715), and TyG index (OR: 2.061, 95% CI: 1.059–2.241) were influencing factors for clinically suspected lower extremity DVT occurrence in patients with intracerebral hemorrhage (*p* < 0.05) (see Table 4).

**Table 3:** Logistic regression analysis variable assignment table.

Variable	Assignment
Age	Continuous variable
Albumin	Continuous variable
D-dimer	Continuous variable
TyG index	Continuous variable
Dependent variable	DVT = 1, Non DVT = 0

**Table 4:** Multivariate logistic regression analysis of clinically suspected lower limb DVT in patients with cerebral hemorrhage.

Influencing factors	β	SE	Wald χ^2^	OR	95%CI	*P*	VIF
Age	0.187	0.042	20.119	1.206	1.111 ~ 1.308	<0.001	2.085
Albumin	−0.292	0.058	25.467	0.747	0.667 ~ 0.836	<0.001	2.166
D-dimer	3.465	1.052	10.842	1.992	1.066 ~ 2.715	0.001	1.975
TyG index	2.087	0.494	17.821	2.061	1.059 ~ 2.241	<0.001	1.862
Constant	−24.132	5.480	19.394	<0.001	~	<0.001	

### 3.5 Construction of a nomogram prediction model for clinically suspected lower extremity DVT occurrence in patients with intracerebral hemorrhage

**Figure 3:**
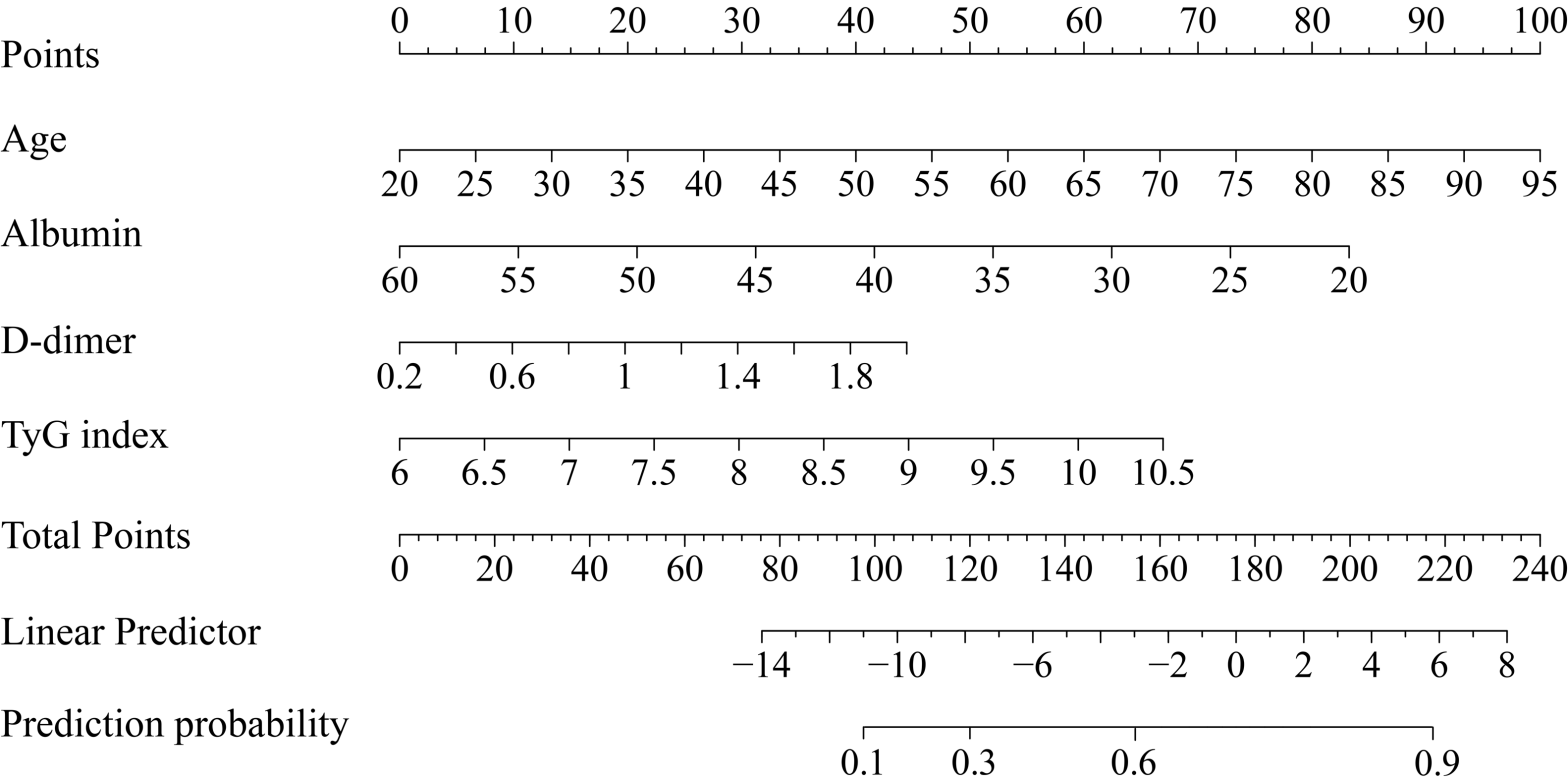
Nomogram prediction model for clinically suspected lower limb DVT in patients with cerebral hemorrhage.

A combined prediction model was constructed based on the *β* coefficients from logistic regression, resulting in the following equation: Logit (P) = −24.132 + 0.187 × Age−0.292 × Albumin + 3.465 × D-dimer + 2.087 × TyG index. Using the RMS package in R software, the prediction model was visualized as a nomogram (Figure 3).

### 3.6 Validation of the nomogram prediction model

**Figure 4:**
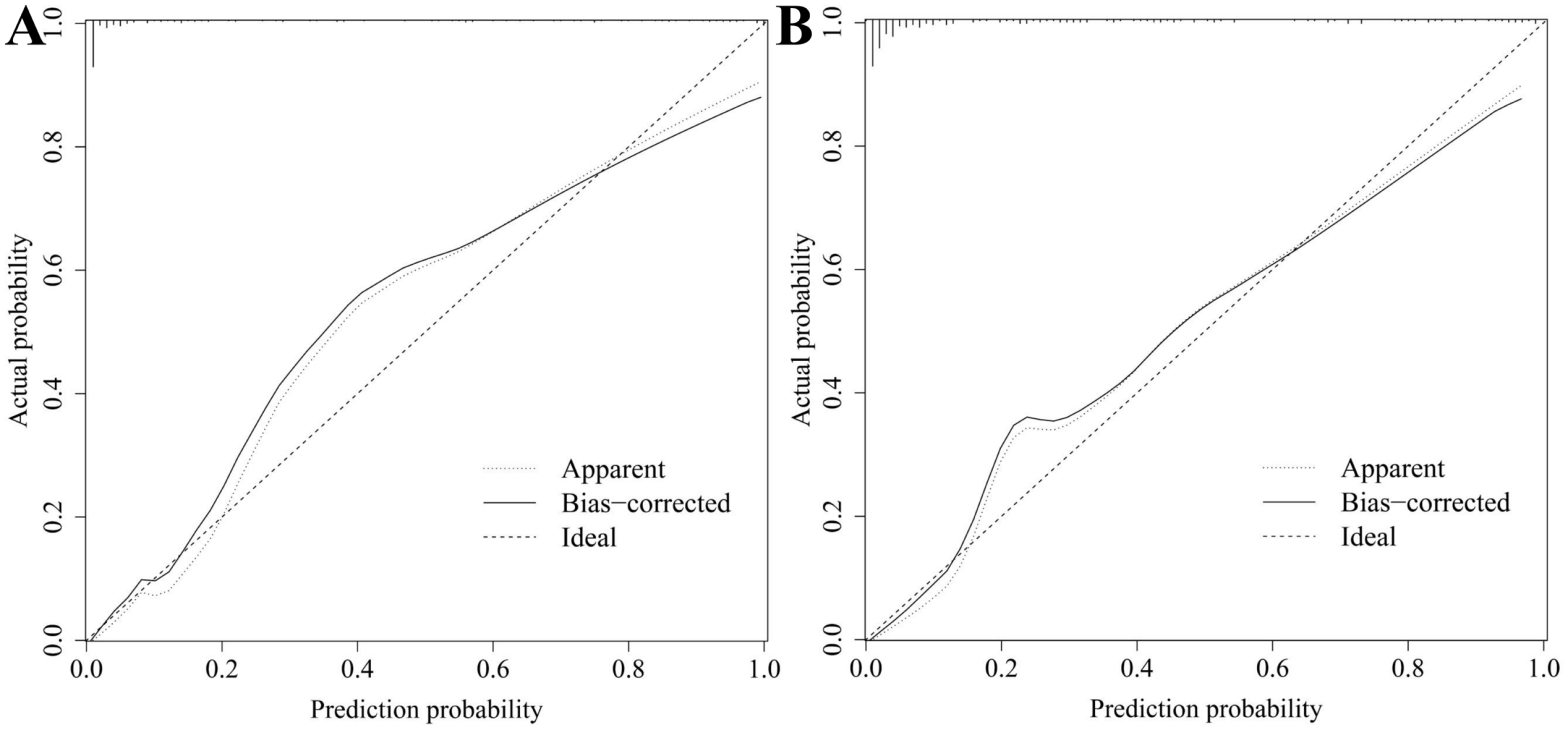
Calibration curve of the nomogram prediction model. **(A)** Training group. **(B)** Validation group.

**Figure 5:**
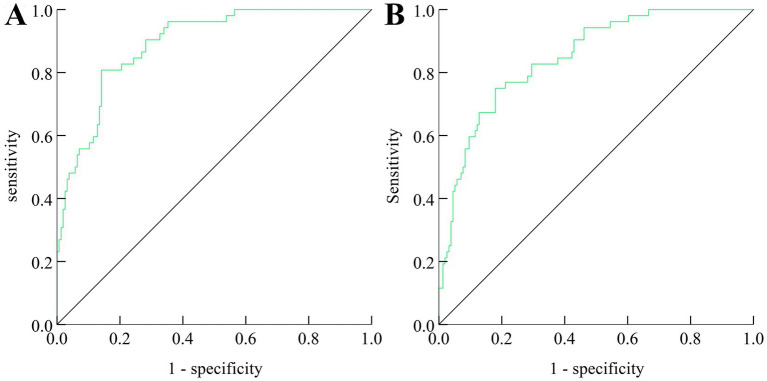
ROC curve of nomogram prediction model. **(A)** Training group. **(B)** Validation group.

**Figure 6:**
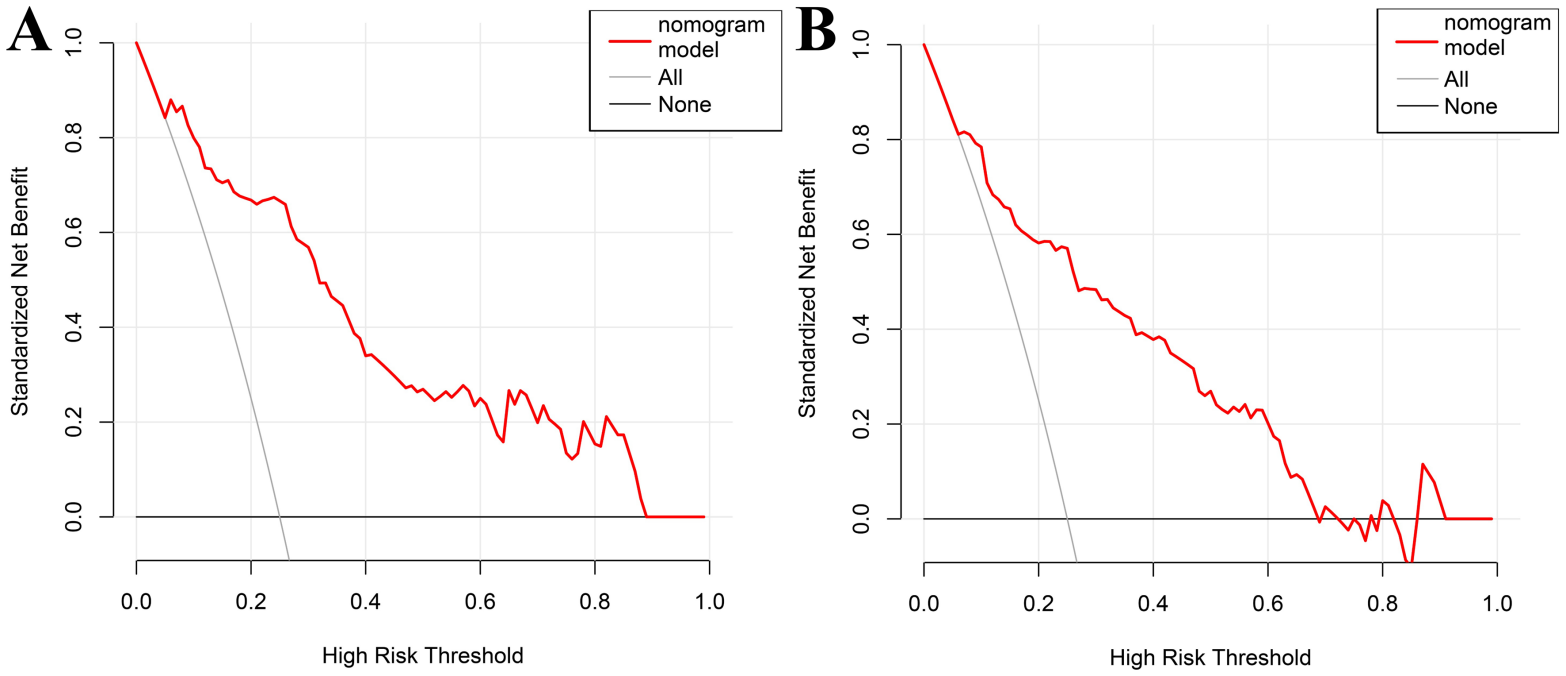
DCA curve of nomogram prediction model. **(A)** Training group. **(B)** Validation group.

The calibration curves of the training group (Figure 4A) and the validation group (Figure 4B) showed Hosmer–Lemeshow χ^2^ statistics of 6.482 and 7.935, with *p* values of 0.627 and 0.592 (>0.05), respectively, indicating no statistically significant difference between predicted values and actual observed values, thus demonstrating good calibration of the model. The ROC curves of the training group (Figure 5A) and the validation group (Figure 5B) showed AUC values of 0.891 (95% CI: 0.845 ~ 0.937) and 0.848 (95% CI: 0.791 ~ 0.905), respectively, indicating good discrimination of the model. The DCA curves of the training group (Figure 6A) and the validation group (Figure 6B) demonstrated that the model provided favorable net clinical benefits within the high-risk threshold probability ranges of 0.07–0.89 and 0.09–0.69, respectively.

## 4 Discussion

Studies have shown that the incidence of DVT during hospitalization in patients with intracerebral hemorrhage is approximately 20–40% (7). In this study, 52 of the 208 patients in the training group developed lower extremity DVT (25.00%), which is consistent with the above findings. Furthermore, in this retrospective study, age, albumin, D-dimer, and TyG index were identified as the best predictors of clinically suspected lower extremity DVT occurrence in patients with intracerebral hemorrhage. A nomogram model was developed and validated for predicting the risk of lower extremity DVT in patients with intracerebral hemorrhage. This nomogram demonstrated a certain predictive performance in this single-center study, with AUCs of 0.891 and 0.848 in the training and validation groups, respectively, and relatively good agreement between predicted and observed probabilities, indicating discrimination and a certain degree of calibration; however, external validation is still required.

This study identified four independent predictive factors. For each one-unit increase in age, the risk of clinically suspected lower extremity DVT occurrence increased by 1.206 times. Qiu et al. (8) similarly found that age, bleeding volume, and surgical status are risk factors for DVT in patients with spontaneous intracerebral hemorrhage. The main explanation is that coagulation capacity increases with age: coagulation factor activity increases, fibrinolytic activity decreases, and blood becomes more prone to clotting, raising the risk of DVT. In addition, venous elasticity and vascular contractility decrease in elderly patients, slowing blood flow, prolonging venous stasis, and increasing the likelihood of DVT formation (9). In this study, lower albumin levels were associated with a higher risk of clinically suspected lower extremity DVT occurrence, a finding consistent with previous research in this field (10). Hypoalbuminemia may increase venous thromboembolism risk through complex mechanisms, including decreased colloid osmotic pressure, increased blood viscosity, imbalance of coagulation and anticoagulation systems, interaction with inflammation affecting endothelial glycoprotein integrity, and promotion of endothelial injury.

Zhang et al. (11) reported that D-dimer is an independent predictor of lower extremity DVT after endovascular treatment of ruptured intracranial aneurysms, a finding similar to ours. Previous studies have noted that elevated D-dimer levels typically indicate a high likelihood of thrombosis (12, 13). D-dimer is a fibrin degradation product formed during coagulation, and elevated levels suggest activation of the coagulation system, fibrin and platelet aggregation, and possible thrombosis. The TyG index, calculated from triglycerides and fasting blood glucose, is a novel marker of insulin resistance, with triglycerides reflecting adipocyte-related resistance and fasting glucose reflecting hepatic insulin resistance (14, 15). Zhang et al. (16) also found that the TyG index has predictive value for DVT in patients with intracerebral hemorrhage, consistent with our findings. Elevated TyG index reflects insulin resistance, which worsens vascular damage and thrombosis, and is closely linked to DVT occurrence. In addition, a previous study (17) showed that intraventricular extension of intracerebral hemorrhage is a marker of severe disease and usually indicates a poorer overall prognosis. In the present study, intraventricular hemorrhage was included as a categorical variable in the univariate analysis. The results showed that there was no statistically significant difference in the incidence of intraventricular hemorrhage between the DVT group and the non-DVT group. This suggests that, in this cohort, intraventricular extension alone was not a direct risk factor for clinically suspected DVT formation, and this finding may be related to the specific inclusion criteria and outcome measures of this study. Diao et al. (18) found that prolonged hospital stay was a risk factor for venous thromboembolism in patients with acute spontaneous intracerebral hemorrhage. Patients with intracerebral hemorrhage, especially those with impaired consciousness or hemiplegia, usually require prolonged bed rest. Prolonged immobilization may slow or even stagnate blood flow, thereby increasing shear stress among blood cells and causing cellular damage, which can activate coagulation factors and increase the risk of DVT (19). However, in the present study, length of hospital stay was considered an outcome variable resulting from the disease rather than a baseline predictor; therefore, it was excluded from the univariate analysis.

Zhang et al. (20) reported that the Caprini score had an AUC of 0.77 for predicting DVT in orthopedic trauma patients. Wen et al. (21) showed that combining the Wells score with the platelet-to-lymphocyte ratio and D-dimer-to-fibrinogen ratio achieved an AUC of 0.951 in predicting DVT in young patients with intracerebral hemorrhage. In our study, the nomogram constructed from age, albumin, D-dimer, and TyG index achieved AUCs of 0.891 and 0.848 in the training and validation groups, respectively, higher than Zhang et al. (20) but lower than Wen et al. (21). These results suggest that predictive performance varies among different scoring systems, but the nomogram in this study showed relatively stable overall performance with a certain degree of discrimination and calibration in the context of a single-center design. Compared with the model constructed by Zhang et al. (16), the present study not only confirmed the importance of the TyG index, but also, for the first time, systematically incorporated D-dimer, which directly reflects coagulation/fibrinolytic activation, and albumin, which reflects nutritional and inflammatory status, into the same prediction model. This multidimensional integration of metabolic, coagulation, and nutritional factors more comprehensively captured the pathophysiological mechanisms underlying clinically suspected DVT and may be one of the reasons why the present model achieved better discrimination. Furthermore, DCA curves indicated a certain degree of clinical applicability. Based on these findings, clinicians are encouraged to use the nomogram to preliminarily identify patients at risk of clinically suspected lower extremity DVT occurrence after intracerebral hemorrhage, particularly those who are older, have elevated D-dimer and TyG index, and have lower albumin levels. Such patients may be prioritized for pharmacological prophylaxis. For patients with temporary immobility or loss of mobility requiring prolonged bed rest, supportive measures such as leg elevation, leg massage, gradient compression stockings, and intermittent pneumatic compression devices may be considered for mechanical thromboprophylaxis. Additionally, the frequency of lower extremity venous ultrasound screening may be increased in these patients to enable early detection of lower extremity DVT. However, external validation of the prediction model was not performed in this study, and whether it has important clinical application value remains to be verified.

However, this study has certain limitations. First, the sample size was limited, and the lack of sufficient variable information restricted the study. Second, as a single-center retrospective study, the findings may not be generalizable to other centers. It should be particularly noted that the specific clinical management protocols within our institution—such as the routine screening frequency for lower extremity DVT, the timing of initiation of mechanical prophylaxis (e.g., intermittent pneumatic compression devices), and the practice standards for pharmacological prophylaxis (e.g., low-dose low-molecular-weight heparin)—may have systematically influenced the detection rate of DVT and may represent unmeasured confounding factors in this study. Differences in clinical practice across institutions may limit the direct generalizability of this prediction model. Third, methodological inconsistencies in defining risk factors mean that their association with DVT in patients with intracerebral hemorrhage remains uncertain. Fourth, all cases included in this study were deep hemorrhages such as basal ganglia and thalamic hemorrhages or non-intraparenchymal hemorrhages such as intraventricular and subdural hemorrhages, and no patients with lobar hemorrhage were included, which may limit the applicability of the model to the lobar hemorrhage population. The clinical characteristics of acute spontaneous lobar hemorrhage differ from those of deep subcortical hemorrhage, as lobar hemorrhage is mainly associated with non-hypertensive mechanisms and is generally associated with a poorer early prognosis. Fifth, the diagnosis of DVT in this study relied on an “ultrasound examination based on clinical suspicion” approach rather than systematic, scheduled ultrasound screening of all enrolled patients. Although this approach is consistent with real-world clinical practice, it may introduce verification bias and thereby systematically underestimate the true incidence of DVT in this cohort. As a result, the prediction model developed in this study essentially predicts the risk of DVT that becomes clinically symptomatic and is subsequently suspected by clinicians. To address these limitations, future prospective, multicenter studies should include larger patient cohorts, expand the range of risk factors, standardize definitions, and further validate and refine the nomogram model.

In conclusion, age, albumin, D-dimer, and TyG index are effective predictors of lower extremity DVT in patients with intracerebral hemorrhage. The nomogram developed in this study demonstrated a certain degree of discrimination, calibration, and net clinical benefit in predicting the risk of clinically suspected lower extremity DVT occurrence, and after external validation, may be of certain significance in helping healthcare providers identify high-risk patients early, support stratified management, and implement targeted interventions to reduce the occurrence of DVT. In future studies, more patients with intracerebral hemorrhage will be included to further validate the generalizability of the model in a mixed cohort comprising both lobar and deep hemorrhages. Before external validation is completed, the findings of this study should be considered preliminary.

## Data Availability

The original contributions presented in the study are included in the article/supplementary material, further inquiries can be directed to the corresponding author.
